# Patchouli alcohol improved diarrhea-predominant irritable bowel syndrome by regulating excitatory neurotransmission in the myenteric plexus of rats

**DOI:** 10.3389/fphar.2022.943119

**Published:** 2022-11-14

**Authors:** Wanyu Chen, Lu Liao, Zitong Huang, Yulin Lu, Yukang Lin, Ying Pei, Shulin Yi, Chen Huang, Hongying Cao, Bo Tan

**Affiliations:** ^1^ Research Centre of Basic Intergrative Medicine, School of Basic Medical Sciences, Guangzhou University of Chinese Medicine, Guangzhou, China; ^2^ Shenzhen Hospital of Shanghai University of Traditional Chinese Medicine, Guangzhou, China; ^3^ College of Integrated Chinese and Western Medicines, Hunan University of Chinese Medicine, Changsha, Hunan, China; ^4^ School of Chinese Materia Medica, Guangzhou University of Chinese Medicine, Guangzhou, China

**Keywords:** patchouli alcohol, irritable bowel syndrome with diarrhea, colonic longitudinal muscle myenteric plexus, excitatory neurons, intestinal motility

## Abstract

**Background and Purpose:** Irritable bowel syndrome (IBS) is usually associated with chronic gastrointestinal disorders. Its most common subtype is accompanied with diarrhea (IBS-D). The enteric nervous system (ENS) modulates major gastrointestinal motility and functions whose aberration may induce IBS-D. The enteric neurons are susceptible to long-term neurotransmitter level alterations. The patchouli alcohol (PA), extracted from *Pogostemonis Herba*, has been reported to regulate neurotransmitter release in the ENS, while its effectiveness against IBS-D and the underlying mechanism remain unknown.

**Experimental Approach:** In this study, we established an IBS-D model in rats through chronic restraint stress. We administered the rats with 5, 10, and 20 mg/kg of PA for intestinal and visceral examinations. The longitudinal muscle myenteric plexus (LMMP) neurons were further immunohistochemically stained for quantitative, morphological, and neurotransmitters analyses.

**Key Results:** We found that PA decreased visceral sensitivity, diarrhea symptoms and intestinal transit in the IBS-D rats. Meanwhile, 10 and 20 mg/kg of PA significantly reduced the proportion of excitatory LMMP neurons in the distal colon, decreased the number of acetylcholine (Ach)- and substance P (SP)-positive neurons in the distal colon and restored the levels of Ach and SP in the IBS-D rats.

**Conclusion and Implications:** These findings indicated that PA modulated LMMP excitatory neuron activities, improved intestinal motility and alleviated IBS-induced diarrheal symptoms, suggesting the potential therapeutic efficacy of PA against IBS-D.

## 1 Introduction

The irritable bowel syndrome (IBS) is a common gastrointestinal disorder affecting physical symptoms and social functioning. Although IBS is not associated with serious complications or mortality, abdominal pain and bloating are often of chronic nature. The pathophysiological mechanisms of IBS remain unknown (Niesler et al., 2021). Several factors have been implicated, including the microbiota, the gut-brain axis (De Palma et al., 2014; Thumann et al., 2019) and psychosocial and environmental factors (Dunn and Adams, 2014; Zhu et al., 2019; van Thiel et al., 2020). The microenvironment of peripheral neurons in the gut wall is presumably involved (Buhner et al., 2014), suggesting the importance local transmitters in the mediation of abdominal pain, discomfort and motility (Knowles et al., 2013). The diagnostic criteria of IBS changed to the Rome IV, which indicated that IBS with diarrhea (IBS-D) is the most common subtype (Ford et al., 2020; Bb et al., 2021).

The digestive system is innervated through its connections with the central nervous system (CNS) and by the enteric nervous system (ENS) within the wall of the gastrointestinal tract (Neunlist & Schemann, 2014). The ENS mainly modulates gastrointestinal motility and functions like secretion, absorption, permeability of epithelial cells and immune activities (Furness & Stebbing, 2018). In the wall of the gut, enteric neurons are susceptible to long-term changes in neurotransmitter levels that ultimately impact the gastrointestinal function (Altaf & Sood, 2008; Neunlist & Schemann, 2014). Acetylcholine (Ach) and substance P (SP) are the principal excitatory neurotransmitters (Clemens et al., 2003). In the gastrointestinal tract, Ach transmits excitatory signals through the receptors expressed by smooth muscle cells. SP can cause contractions of the smooth muscles, promoting intestinal peristalsis and provoking diarrhea (Smith et al., 2007).

The patchouli alcohol (PA) is a tricyclic sesquiterpene extracted from *Pogostemonis Herba* (Cho et al., 2015). PA has been associated with different pharmacological effects, including anti-inflammatory, antibacterial and anticancer properties (Hu et al., 2017). In addition, PA regulates colonic smooth muscle activity through cholinergic and non-cholinergic nerves. Zhou et al. (2018) reported that PA might potentially treat IBS-D by influencing the neurotransmitter release in the ENS. Although PA may affect the release of gastrointestinal transmitters through multiple pathways and targets, whether it would be of benefit for IBS-D is unknown.

## 2 Methods

### 2.1 Animals and treatments

Fifty-four male Sprague-Dawley rats were randomly divided into six model groups and three control groups (*n* = 6 per group). Initially, rats in model groups were stressed between 4:00 and 6:00 p.m. for 14 days. The upper limbs, shoulders and chest were bound with a medical elastic mesh bandage, the rats were restricted from scratching the head and face, but other activities were not restricted. After modeling, the rats were divided into three model groups and three PA groups with low (5 mg/kg), medium (10 mg/kg), and high (20 mg/kg) doses. PA groups received gavage administration for 2 weeks, while control and model groups received an equal volume of Tween 80.

### 2.2 Visceral sensitivity assessment

Behavioral responses to colorectal distention (40 and 60 mmHg) were assessed by measuring the abdominal withdrawal reflex (AWR) on day 14, day 21, and day 28 after modeling, as previously described (Williams et al., 1988). Briefly, rats were anesthetized with diethyl ether after fasting for 12 h. Then, a catheter was inserted through the anus and the outer end of the distention balloon was secured by taping the attached tubing to the rat’s tail. After adaption for 1 h, animal responses to colorectal distention were blindly examined by two investigators.

### 2.3 Evaluation of defecation

On day 14, day 21, and day 28 after modeling, rats were placed individually in clean cages with food and water *ad libitum*. The defecation area was monitored within 4 h and evaluated with the ImageJ Software.

### 2.4 Intestinal transit

After modeling, rats fasted for 24 h with free access to water on day 14, day 21, and day 28. Then, they received 1 ml of powdered carbon (Activated Carbon Powder) *via* gastric gavage. Thirty minutes later, rats were euthanized and the bowel was removed. The length of the intestinal tract from the gastroduodenal junction to the anus and the length of the tract containing the carbon were measured. The intestinal transit rate was calculated through the following equation: length of the intestinal tract containing the carbon/length of the intestinal tract × 100%.

### 2.5 Tissues preparation

The colon was dissected and placed in ice-cold Krebs solution. The Krebs solution was pretreated with 95% oxygen and 5% CO_2_ for at least 30 min. Then, the colon was cut into several segments and flushed with ice-cold Krebs solution to clean the fecal matter. The longitudinal muscle myenteric plexus (LMMP) was separated and placed on polylysine glass slides. Cold paraformaldehyde (PFA) was added in 0.1 M of sodium phosphate buffer saline (PBS) and LMMP was bathed for 4–6 h at 4°C. After fixture, the samples were washed with PBS and cryoprotected overnight with 30% sucrose dissolved in PBS at 4°C. The longitudinal muscle strips were separated with the same procedure and placed at liquid nitrogen for RNA extraction.

### 2.6 Immunohistochemistry

After cryoprotection, LMMP was rinsed with PBS and cut into 10 * 10 mm pieces. Tissue wholemounts were placed in 5% bovine serum albumin and 0.3% Triton X-100 in PBS for 2 h at room temperature. Later, LMMP samples were exposed to primary antibodies diluted in PBS at 4°C for 21–24 h. The wholemounts were washed with PBS supplemented with 0.05% tween-20 (TPBS) for 5 min and rinsed three times with PBS (5 min each time). Then, the samples were incubated with secondary antibodies for 2 h at room temperature. After incubation, tissue wholemounts were rinsed with TPBS for 15 min, washed with PBS (5 min each time) and coverslipped with fluorescence decay-resistant medium. Concerning choline acetyltransferase (ChAT) and SP immunohistochemistry, tissue wholemounts were heated by water-bath for antigen retrieval. Briefly, samples were placed in 0.01 M citrate buffer (pH 6.0) and heated for 10 min at 100°C. After being washed three times with PBS, the samples were cut into pieces and blocked in 10% donkey serum albumin and 0.3% Triton X-100 in PBS. Primary and secondary antibodies used in this study are provided in Table 1.

**TABLE 1:** Antibodies used for immunohistochemistry.

Antigen	Host	Code	Dilution	Source
HuC/HuD	Rabbit	ab184267	1:500	Abcam
ChAT	Goat	AB144P	1:100	Chemicon
Substance P	Rat	MAB356	1:200	Chemicon
Mouse IgG	Goat Alexa Fluor 488	1010–30	1:100	SouthernBiotech
Rabbit IgG	Goat Alexa Fluor 555	4010–32	1:200	SouthernBiotech
Goat IgG	Donkey Cy3	705–165–147	1:100	Jackson
Rabbit IgG	DyLight 405	711–475–152	1:100	Jackson
Rat IgG	Donkey Alexa Fluor 488	712–545–153	1:100	Jackson

Note: ChAT, choline acetyltransferase; IgG, immunoglobulin G; Cy3, indocarbocyanine.

### 2.7 Quantitative analysis

The quantitative analysis for immunoreactive neurons was performed according to the neuronal density (neurons/mm^2^). Images were obtained using a 10X microscope objective. The ImageJ software was used to calculate the proportion of ChAT-positive and SP-positive neurons.

### 2.8 Morphological analysis

The morphometric analysis of HuC/D-immunoreactive neurons was performed in an area (μm^2^) with 100 neuronal cell bodies. Regarding the SP-immunoreactive neurons, an average area of 400 nerve varicosities from each animal (2,400/group) was used for morphometric analysis.

### 2.9 The mRNA quantification

The total RNA was extracted from LMMP preparations through the TRIzol Reagent method (GIBCO, US). The concentration of mRNAs was evaluated by quantitative polymerase chain reaction (qPCR) analysis. The qPCR was performed using CFX 96 Real-Time Detection System (Bio-Rad, Germany). The concentration was normalized for RNA loading using GAPDH primers. Sequences of the PCR primers are listed in Table 2.

**TABLE 2:** Primer sequences.

Gene	Forward primer (5′–3′)	Reverse primer (5′–3′)
SP	TTA​ATG​GGC​AAA​CGG​GAT​GC	GCG​CTT​CTT​TCA​TAA​GCC​ACA​G
ChAT	TTG​TGC​AAG​CCA​TGA​CTG​AC	ATG​GTT​GTC​AAT​GGC​CAT​GC
GAPDH	TGA​GCA​TCT​CCC​TCA​CAA​TTC​C	TTT​TTG​AGG​GTG​CAG​CGA​AC

### 2.10 Western blot

The LMMP layers were frozen in liquid nitrogen and stored at −80°C until processed. Then, cell lysis was performed with ice-cold RIPA lysis buffer and protease inhibitor cocktail. The total protein concentrations in the extracts were measured with a BCA protein assay kit (Pierce). The membranes were blocked for 1 h at room temperature with nonfat dry milk in Tris-buffered saline (TBS) supplemented with 0.1% tween-20 and incubated overnight with primary antibodies at 4°C. After repeated washing, the membranes were incubated with a horseradish peroxidase-conjugated anti-rabbit secondary antibody at room temperature for 2 h and visualized with a chemiluminescence substrate. Primary and secondary antibodies used in this study are provided in Table 3.

**TABLE 3:** Antibodies used for western blot.

Antigen	Host	Code	Dilution	Source
ChAT	Goat	AB144P	1:1000	Chemicon
Substance P	Rat	MAB356	1:1000	Chemicon
Rabbit anti-Goat IgG (h + l)	HRP	FDR007	1:5000	Fudebio
Goat anti-Rat IgG (h + l)	HRP	FDM007	1:5000	Fudebio

Note: ChAT, choline acetyltransferase; IgG, immunoglobulin G; Cy3, indocarbocyanine.

### 2.11 Statistical analysis

Data are presented as mean ± standard deviation (SD). Group comparisons were performed using the one-way ANOVA test. Values of *p* < 0.05 were considered statistically significant. Tukey’s post hoc test was then used to compare significant differences between groups. Statistical analysis was performed using GraphPad Prism software.

### 2.12 Patchouli alcohol

Patchouli alcohol (purity >99%) was kindly provided by the Mathematical Engineering Academy of Chinese Medicine, and the quality of PA was confirmed by melting point, infrared spectroscopy, ^1^H and ^13^C NMR, and mass spectrometry. DMSO was used to dissolve PA. DMSO <0.5% in all experiments.

## 3 Results

### 3.1 Patchouli alcohol improved intestinal symptoms in Irritable bowel syndrome with diarrhea rats

#### 3.1.1 Visceral sensitivity

**FIGURE 1:**
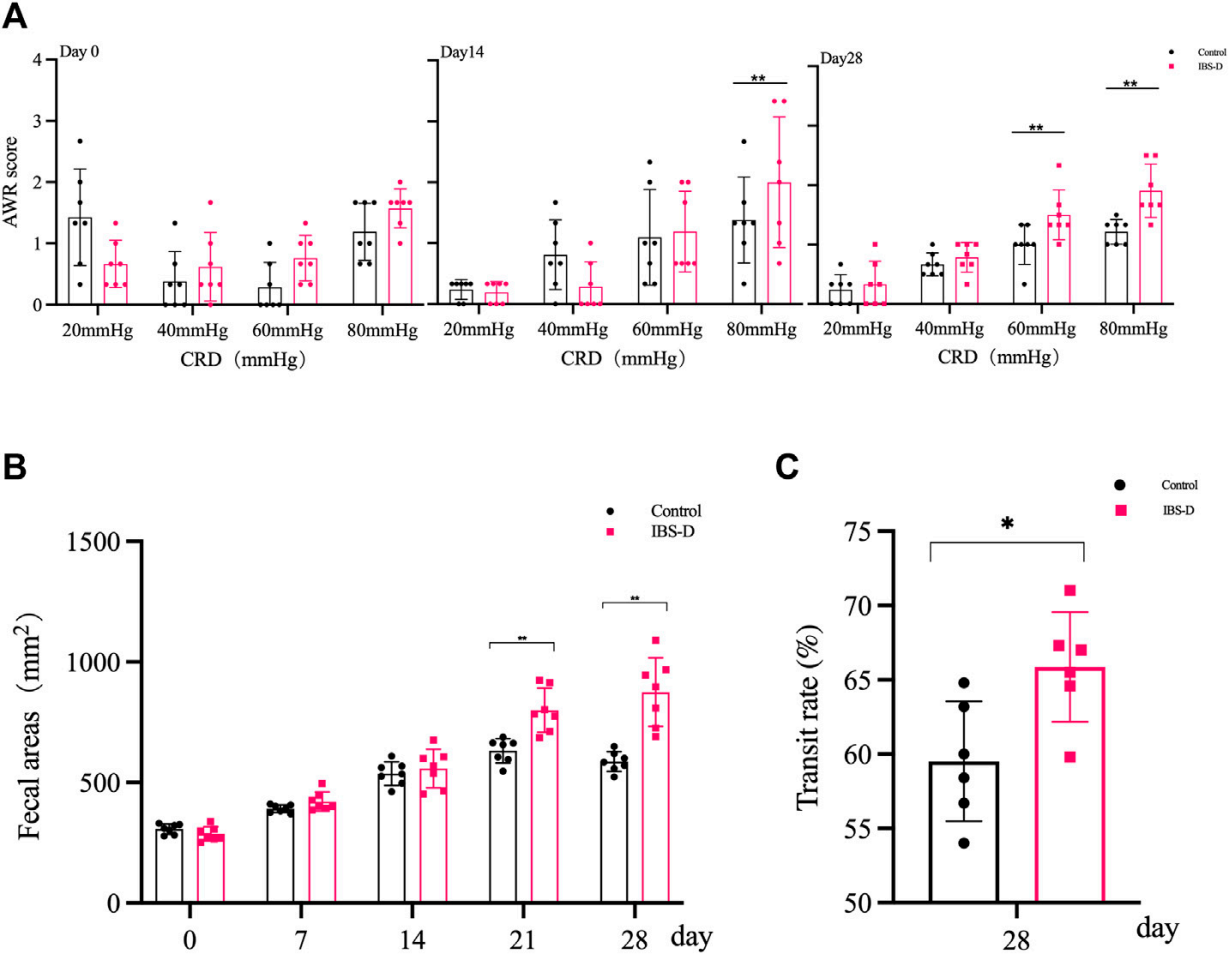
Evaluation of the IBS-D model. **(A)** Abdominal withdrawal reflex assessment in IBS-D rats. The AWR scores were used to assess visceral sensitivity in IBS-D rats at baseline, day 14 and day 28. **(B)** The defecation area evaluated diarrhea symptoms at baseline, day 7, day 14, day 21, and day 28 in different groups. **(C)** The intestinal transit rate revealed the changes in colon motility among model groups. Data are presented as mean ± SD (*n* = 6). **p < 0.01 vs. controls, *p < 0.05 vs. controls.

**FIGURE 2:**
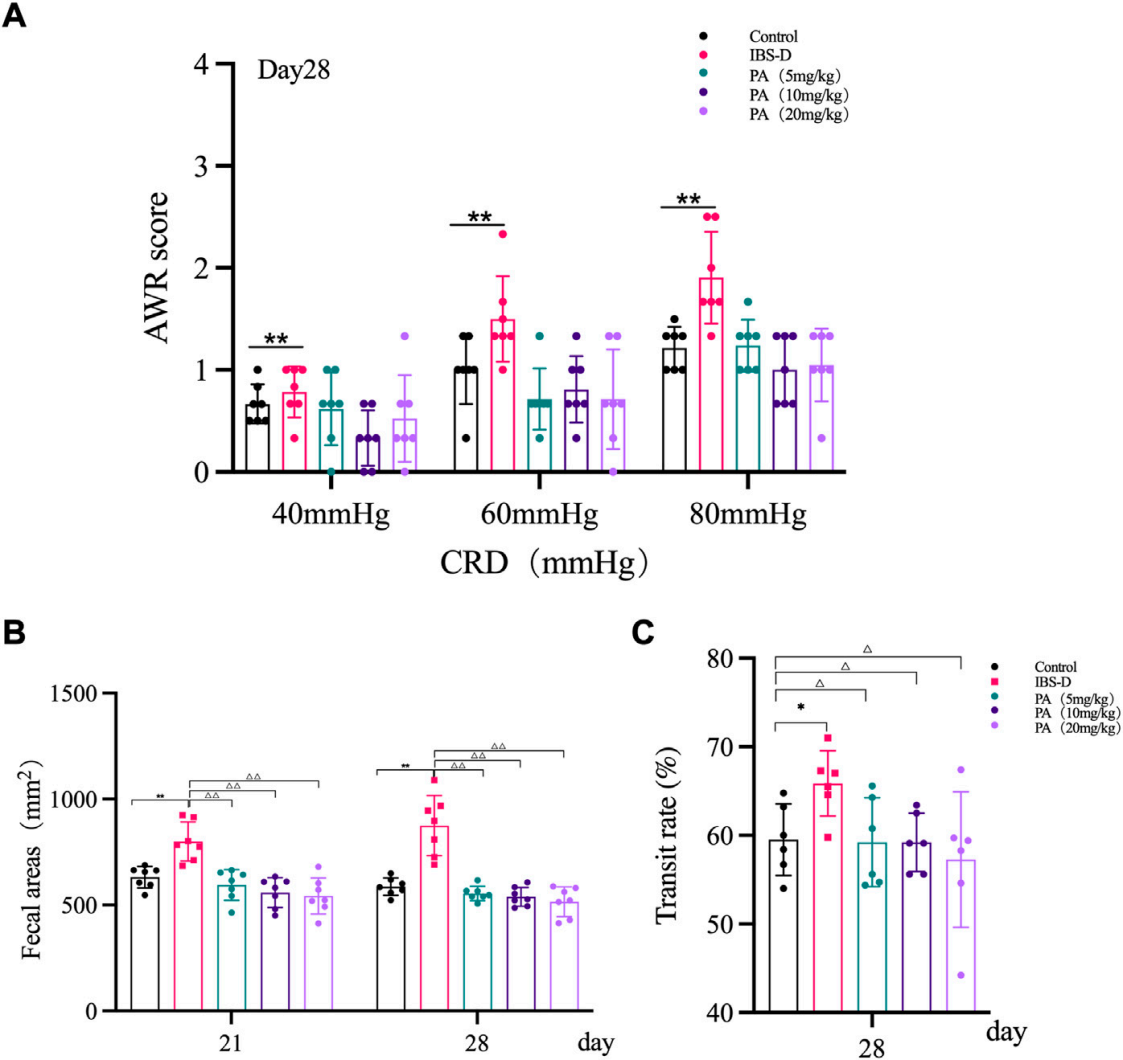
Effects of patchouli alcohol (PA) in IBS-D rats. **(A)** Abdominal withdrawal reflex assessment in different groups after PA. The AWR scores were used to assess visceral sensitivity at day 21 and day 28 after PA. **(B)** The defecation area evaluated diarrhea symptoms at day 7 and day 14 after PA. **(C)** The intestinal transit rate revealed the changes in colon motility among PA groups. Data are presented as mean ± SD (*n* = 6). *p < 0.05, **p < 0.01, vs. controls; ^△^ *p* < 0.05, ^△△^ *p* < 0.01, vs. models.

As shown in Figure 1A, the AWR scores of the model group were significantly increased at day 14 and day 28 compared to the control group (*p* < 0.01), indicating the occurrence of visceral hypersensitivity. As shown in Figure 2A, at day 28 the AWR scores of each PA group were significantly reduced compared to the model group (*p* < 0.05 and *p* < 0.01).

#### 3.1.2 Diarrhea symptoms

As shown in Figure 1B, the defecation area in the model group was significantly increased at day 21 and day 28 compared to the control group (*p* < 0.01), indicating that the defecation frequency increased in the model group. As shown in Figure 2B, the defecation area in each PA group significantly decreased compared to the model group (*p* < 0.01).

#### 3.1.3 Intestinal transit

As shown in Figure 1C, the intestinal transit rate was faster in the model group than the control group. The rate in the model group was 65.9 ± 0.87%, while the rate in the control group was 59.5 ± 4.0% (*n* = 6, *p* = 0.032). As shown in Figure 2C, the intestinal transit rate significantly decreased after treatment with PA.

### 3.2 Patchouli alcohol improved intestinal motility by modulating excitatory neurotransmitters

#### 3.2.1 Substance P

**FIGURE 3:**
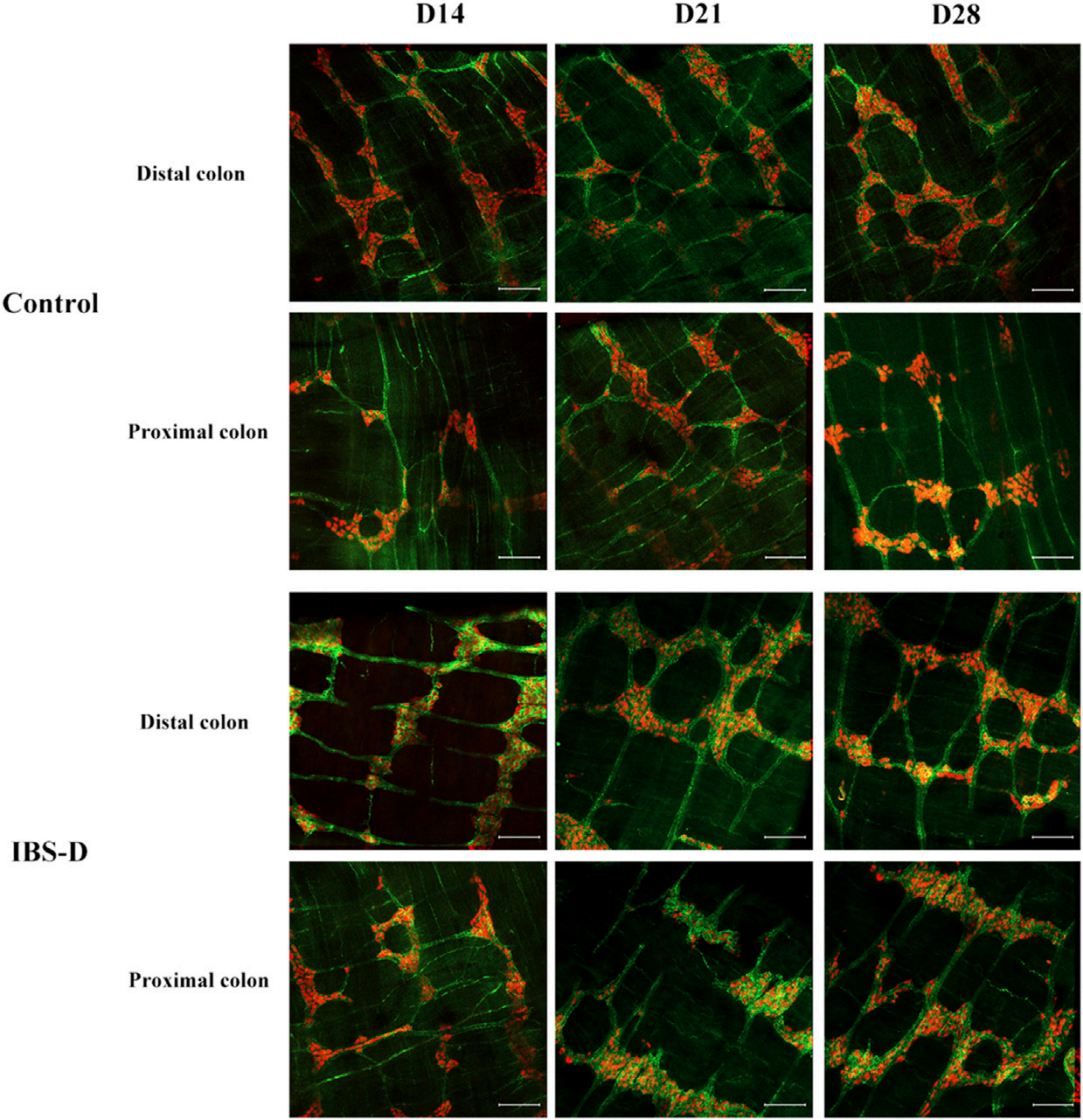
Substance P (SP) was altered in IBS-D rats. SP proteins photographed by laser confocal microscopy. Immunofluorescence results of SP positive neurons in the proximal and distal colon of IBS-D rats at day 14, day 21, and day 28. HuC/D (Red), SP(Green). Scale Bar = 100 μm.

**FIGURE 4:**
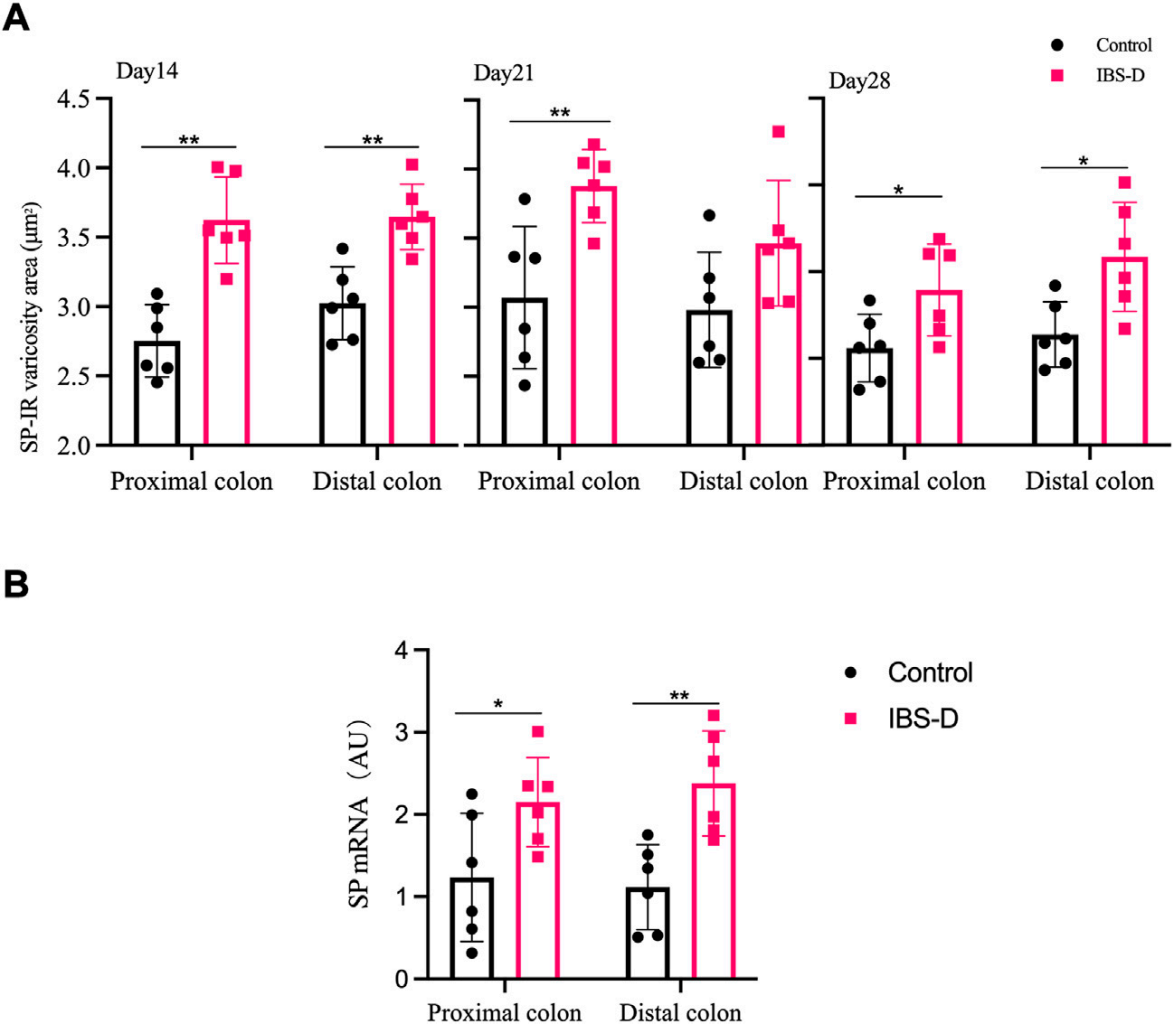
**(A)** SP-immunoreactive varicosities in the distal and proximal colon. **(B)** SP mRNA expression in model groups. RNA was isolated with TRIzol reagent and qRT-PCR was utilized to analyze the gene expression of SP. The GAPDH gene was defined as internal reference. Data were presented as mean ± SD (*n* = 6). *p < 0.05, **p < 0.01, vs. controls.

**FIGURE 5:**
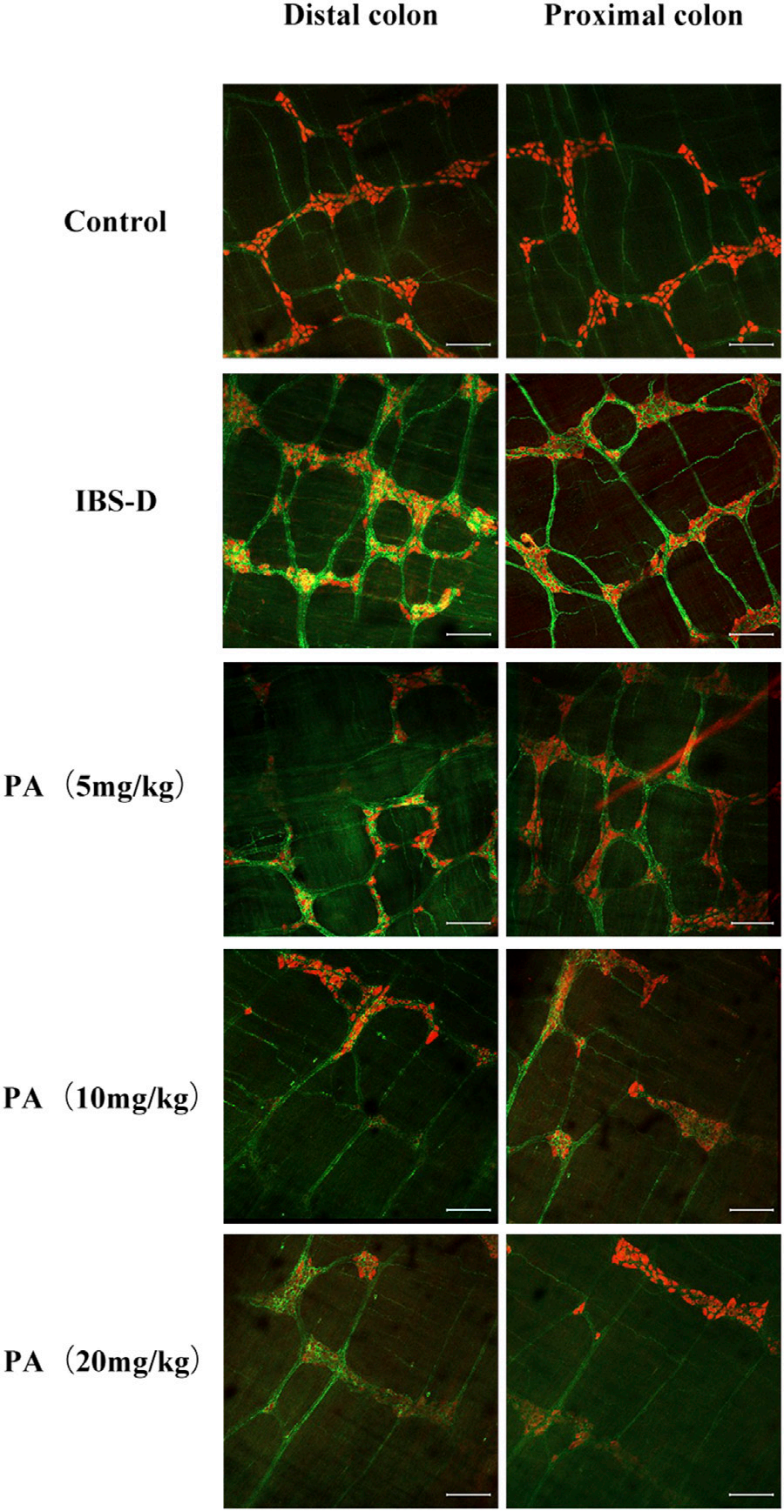
Effects of patchouli alcohol (PA) on substance P (SP). SP proteins photographed by laser confocal microscopy. Immunofluorescence results of SP positive neurons in the proximal and distal colon of PA groups. HuC/D (Red), SP(Green). Scale Bar = 100 μm.

**FIGURE 6:**
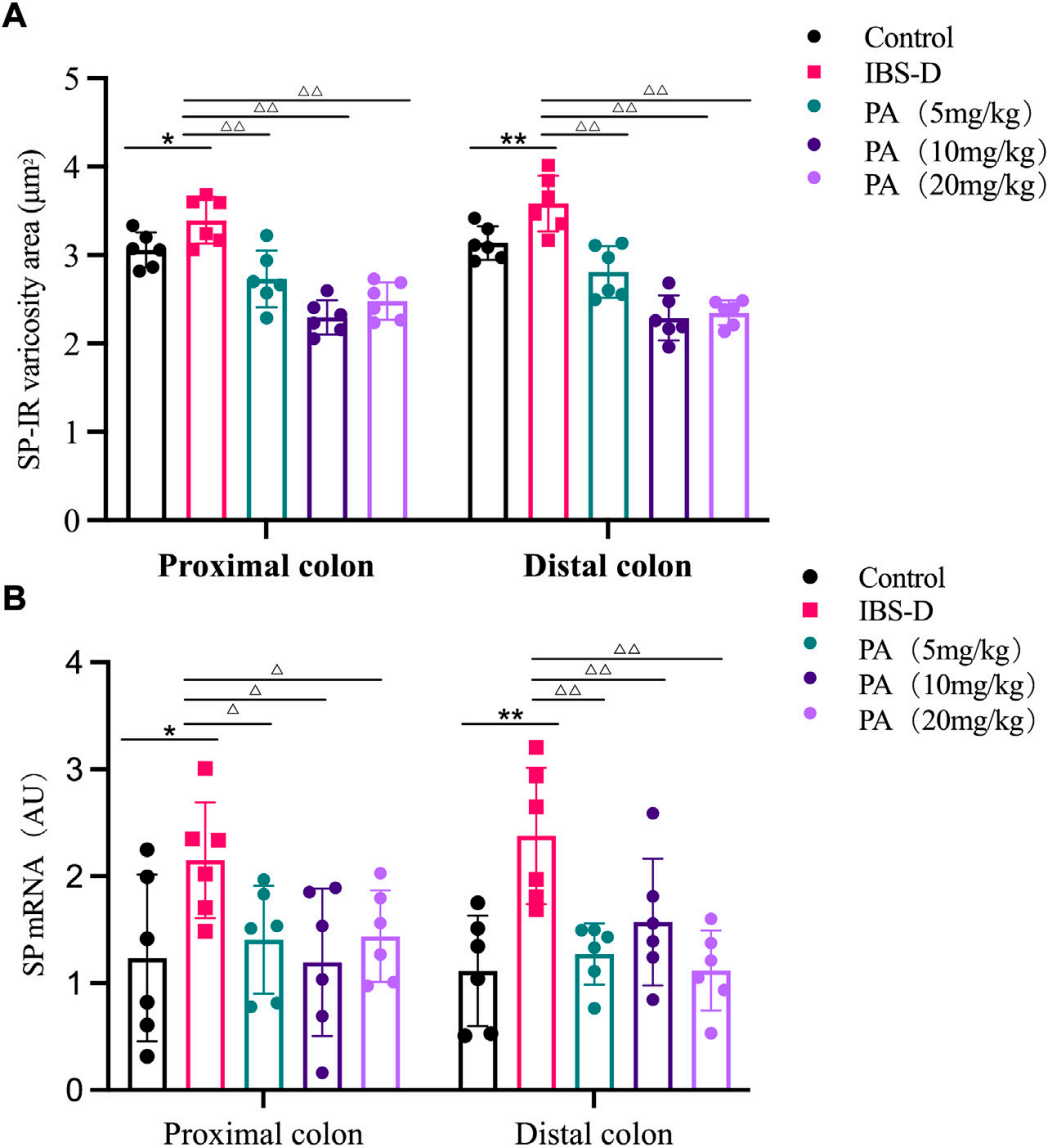
**(A)** SP-immunoreactive varicosities in the distal and proximal colon of PA groups. **(B)** SP mRNA expression in PA groups. RNA was isolated with TRIzol reagent and qRT-PCR was utilized to analyze the gene expression of SP. The GAPDH gene was used as internal reference. Data are presented as mean ± SD (*n* = 6). *p < 0.05, **p < 0.01, vs. controls. ^△^ *p <* 0.05, vs. models, ^△△^ *p* < 0.01, vs. models.

As shown in Figures 3, 4A, immunofluorescence results showed that SP-immunoreactive neurons were significantly increased in the model group compared to the control group (*p* < 0.05). As shown in Figure 4B, the qRT-PCR analysis revealed significantly increased SP expression in the distal and proximal colon of the IBS-D group compared with the control group (*p* < 0.05). As shown in Figure 5, Figure 6A, after treatment with PA for 14 days, the area of SP-immunoreactive varicosities in the distal and proximal colon was significantly decreased in the PA group compared with the IBS-D group (*p* < 0.01). As shown in Figure 6B, the expression of SP mRNA in the distal and proximal colon was significantly decreased in the PA group compared with the IBS-D group (*p* < 0.05).

As shown in Figures 11, 12A, western blot revealed a specific band at approximately 40 kDa in LMMP preparations, representing a post-translational modification of SP. The expression of SP in the proximal colon of the model group was significantly increased compared with the control group (*p* < 0.05). The expression of SP in the distal colon was not significantly elevated. After administration of PA for 14 days, SP levels were downregulated in the distal and proximal colon.

#### 3.2.2 Choline acetyltransferase

**FIGURE 7:**
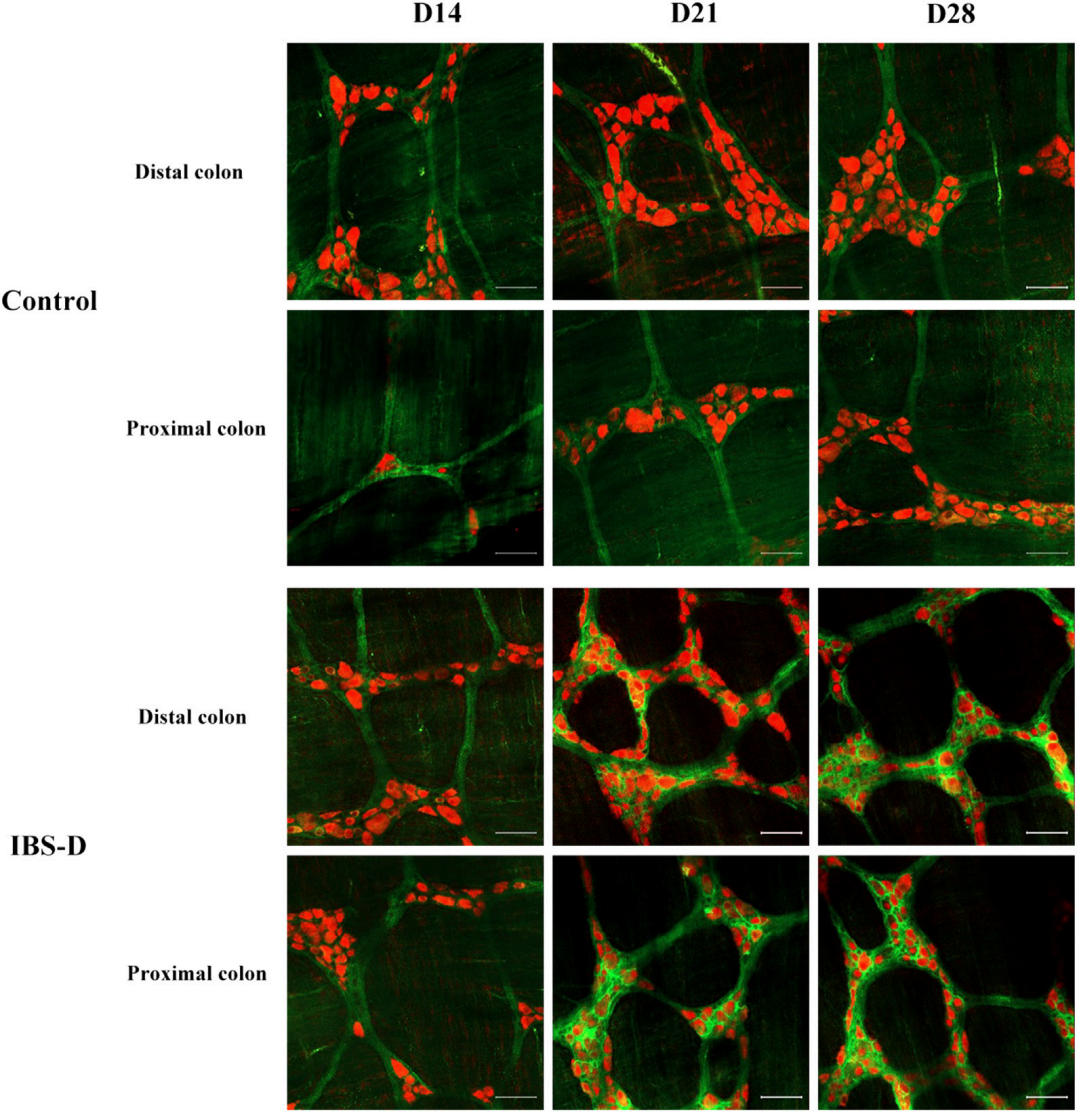
Choline acetyltransferase (ChAT) is altered in IBS-D rats. ChAT proteins photographed by laser confocal microscopy. Immunofluorescence results of ChAT positive neurons in the proximal and distal colon of IBS-D rats at day 14, day 21, and day 28. HuC/D (Red), ChAT (Green). Scale Bar = 50 μm.

**FIGURE 8:**
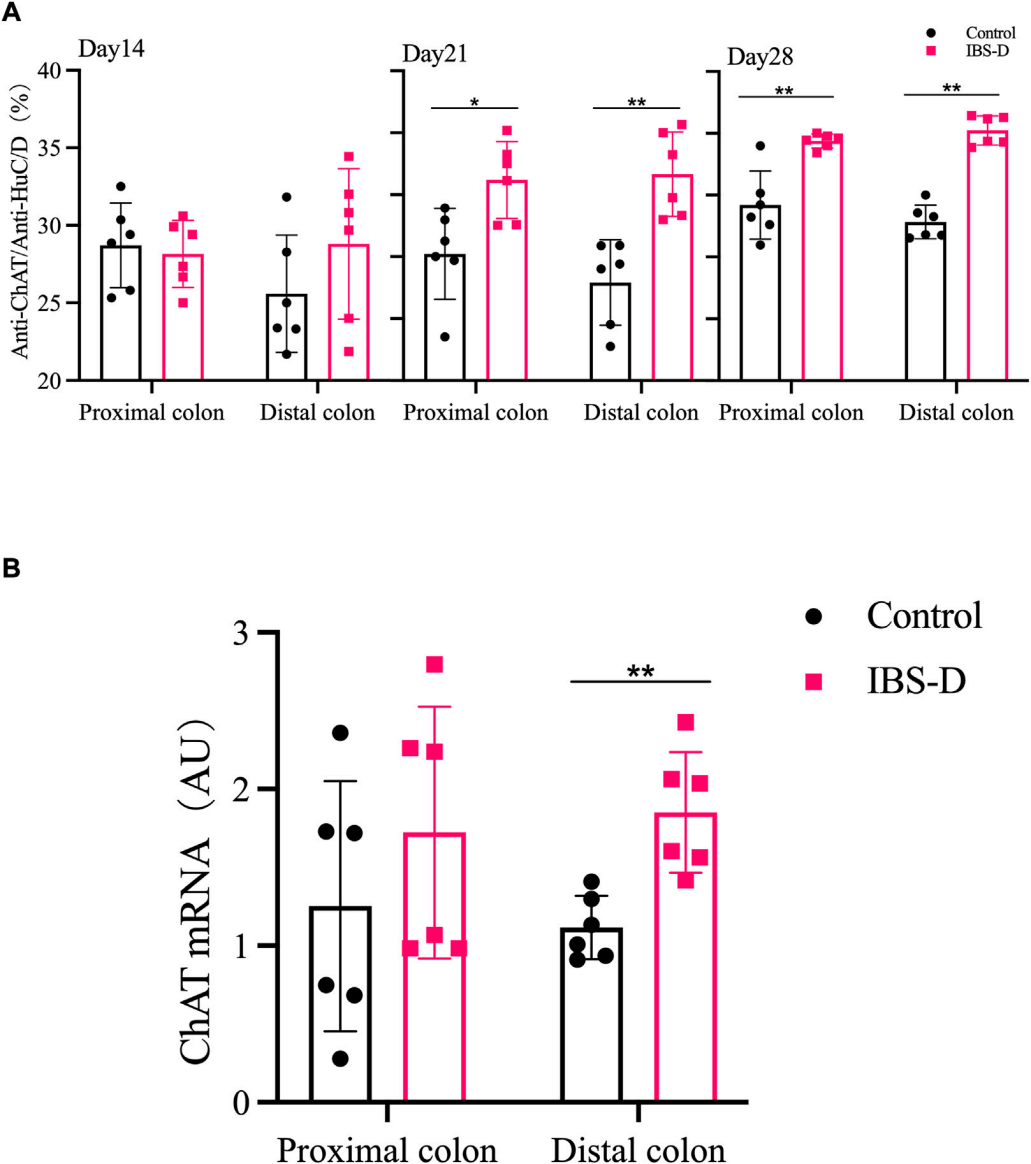
**(A)** The proportion of ChAT positive neurons in the distal and proximal colon. **(B)** ChAT mRNA expression in model groups. RNA was isolated with TRIzol reagent and qRT-PCR was used to analyze the gene expression of ChAT. The GAPDH gene was used as internal reference. Data are presented as mean ± SD (*n* = 6). *p < 0.05, **p < 0.01, vs. controls.

**FIGURE 9:**
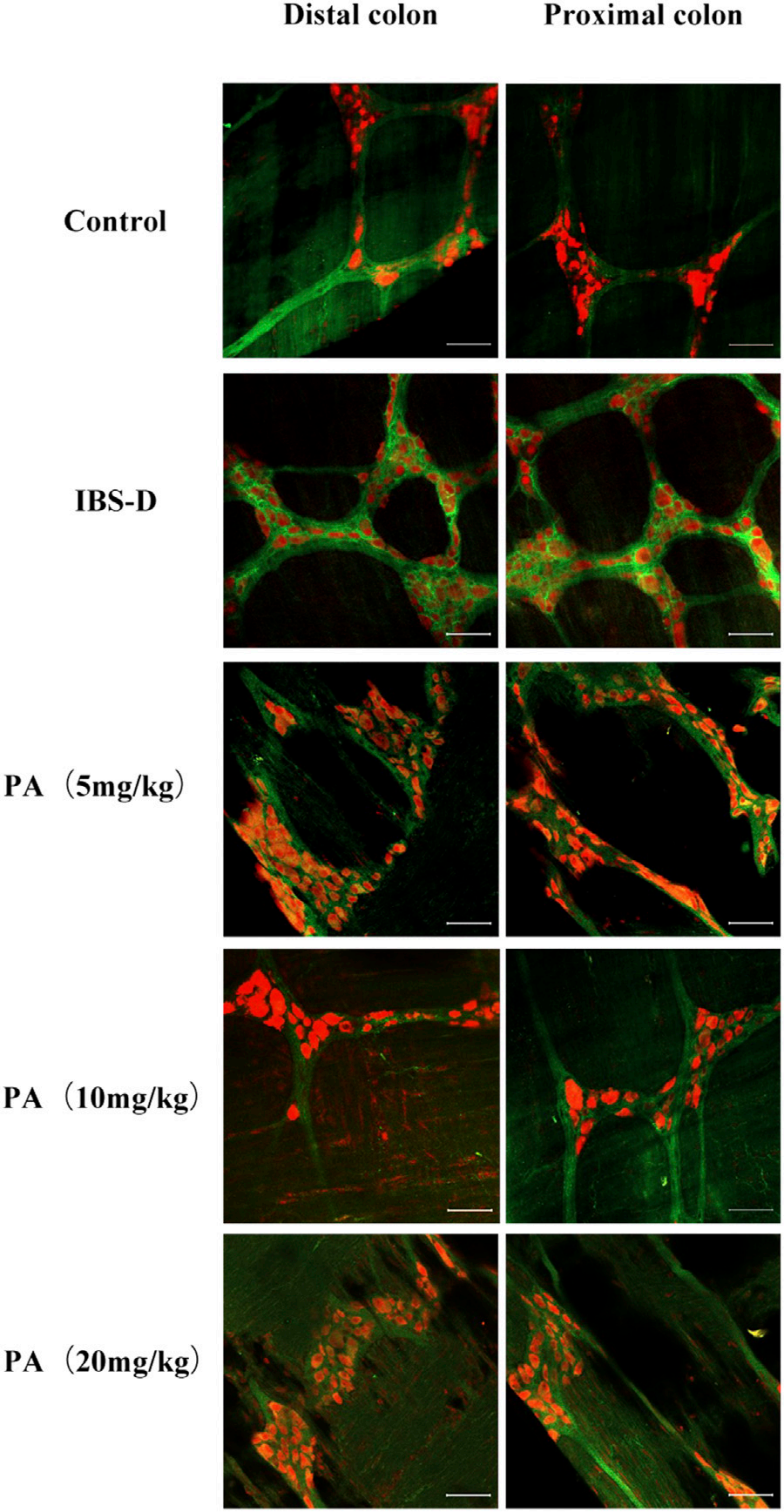
Effects of patchouli alcohol (PA) on choline acetyltransferase (ChAT). ChAT proteins photographed by laser confocal microscopy. Immunofluorescence results of ChAT positive neurons in the proximal and distal colon of PA groups. HuC/D (Red), ChAT (Green) Scale Bar = 50 μm.

**FIGURE 10:**
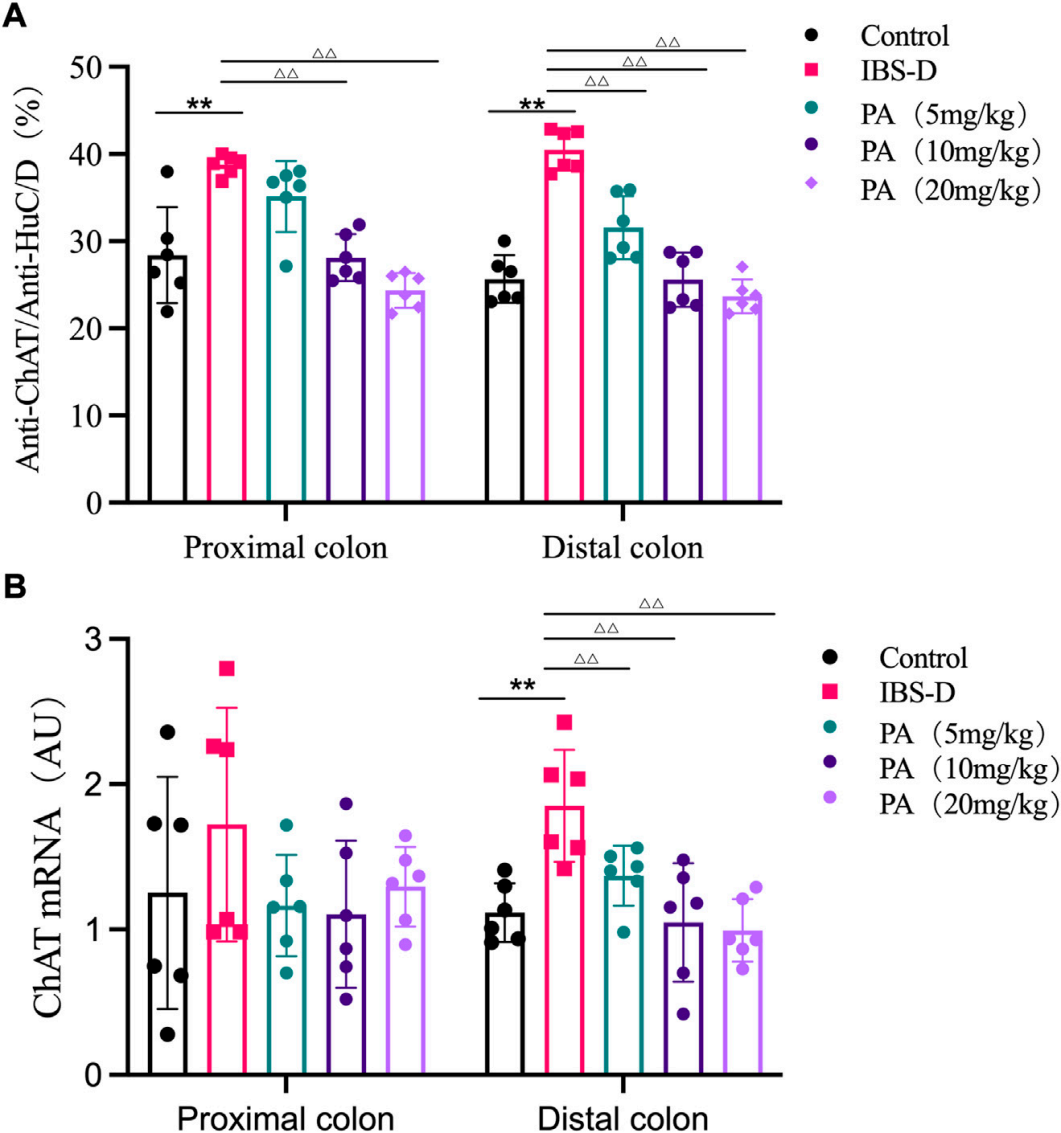
**(A)** The proportion of ChAT positive neurons in the distal and proximal colon of PA groups. **(B)** ChAT mRNA expression in PA groups. RNA was isolated with TRIzol reagent and qRT-PCR was utilized to analyze the gene expression of ChAT. The GAPDH gene was used as internal reference. Data are presented as mean ± SD (*n* = 6). *p < 0.05, **p < 0.01, vs. controls. ^△^ *p <* 0.05, vs. models, ^△△^ *p* < 0.01, vs. models.

As shown in Figures 7, 8A, the proportion of ChAT positive neurons in the distal colon of IBS-D rats was significantly increased (*p* < 0.01). At the same time, there was no significant difference in the proximal colon between the two groups. As shown in Figure 8B, the qRT-PCR analysis revealed significantly increased ChAT expression in the distal colon of IBS-D rats (*p* < 0.05). As shown in Figure 9 and Figure 10A, the proportion of ChAT positive neurons in the distal colon was significantly decreased after treatment with PA (*p* < 0.01), and as shown in Figure 10B, ChAT mRNA expression levels was significantly decreased in the PA group compared with the IBS-D group (*p* < 0.05). On the contrary, the expression of ChAT in the distal colon of IBS-D rats increased (*p* < 0.05). The expression of ChAT in the proximal colon was not significantly elevated. After administration of PA for 14 days (D28), the expression of ChAT in the proximal colon decreased.

**FIGURE 11:**
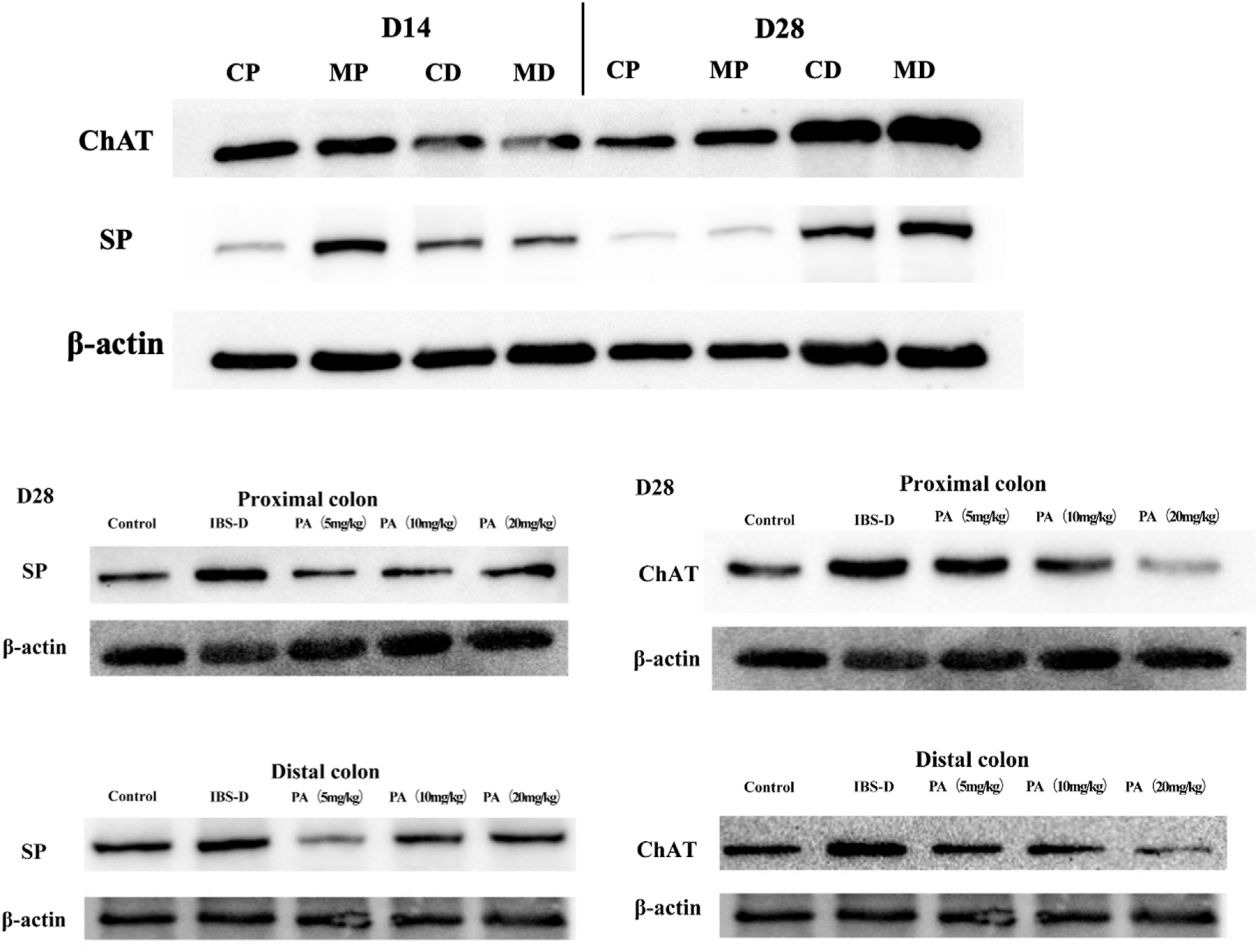
Effects of patchouli alcohol (PA) on substance P (SP) and choline acetyltransferase (ChAT) protein expression (SP and ChAT bands). SP and ChAT proteins were quantified by western blot. CP = proximal colon in control groups, CD = distal colon in control groups, MP = proximal colon in model groups, MD = distal colon in model groups.

**FIGURE 12:**
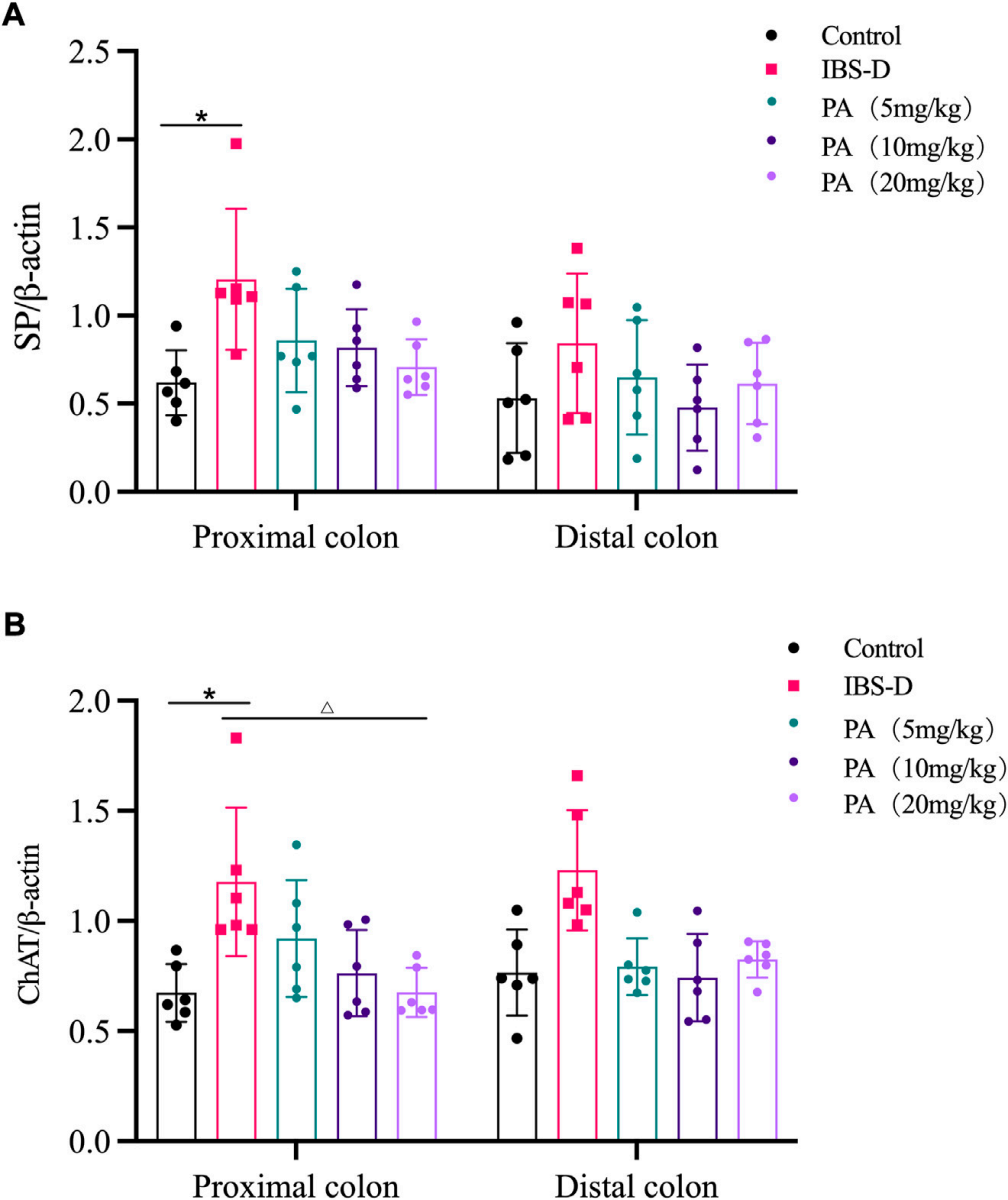
Effects of patchouli alcohol (PA) on substance P (SP) and choline acetyltransferase (ChAT) protein expression. SP and ChAT proteins were quantified by western blot. β-actin was used as internal reference. **(A)** SP protein in the distal and proximal colon of PA groups. **(B)** ChAT protein in the distal and proximal colon of PA groups. Data are presented as mean ± SD (*n* = 6). *p < 0.05, vs. controls. ^△^ *p <* 0.05, vs. models.

As shown in Figures 11, 12B, western blot found a significant difference regarding the expression of ChAT between the high dose group and the model group after PA administration (*p* = 0.003). A significant difference was also found between the model group and the control group (*p* = 0.003). At D28, the expression of ChAT in the distal colon of high, medium and low dosing groups was significantly downregulated (*p* = 0.001, *p* < 0.01 and *p* < 0.01, respectively). High dose of PA treatment significantly decreased ChAT protein expression in the proximal colon comparing to the model group.

## 4 Discussion

Different physiological activities of the intestine depend on neurons. The ENS is a critical controller of the innervation of the large intestine (Bayliss & Starling, 1901), but its relevance in gastrointestinal diseases has largely been overlooked (Furness, 2012; Knowles et al., 2013; Spencer & Hu, 2020). Previous studies identified the relationship between intestinal activity and neurons in different regions of the colon (Costa et al., 2013). Li et al. (2019) designed a Ca^2+^ imaging approach revealing that different regions of the gut exhibited specific motility patterns regulated by myenteric neurons. Hibberd et al. (2018) found that optogenetic control of enteric neurons can increase gut motility and fecal output. However, local effects of myenteric neurons on intestinal function have been insufficiently investigated in drug development. In this study, we focused on the neuronal activities of the myenteric plexus, which innervates the longitudinal and circular muscles in the intestine. We used a previously implemented technique isolating LMMP preparations to observe the morphological changes of the entire colon (Huang et al., 2021). Immunoreactivity for ChAT and SP identified the corresponding neurons in the myenteric plexus (Sang & Young, 1996; Sang et al., 1997). The aim of the present study was to investigate the ENS remodeling in rats with IBS-D and the potential mechanisms of PA for IBS-D treatment. In the wrap-restraint stressed IBS-D rats, the total number of neurons increased in the myenteric plexus of the distal colon, as indicated by an increased number of varicosities and an augmented expression of ChAT and SP. Previous studies found that stress increased colonic motility in animals and human volunteers (Williams et al., 1988). In animal models, diarrhea associated with stress may be related to a local imbalance of neurotransmitters. We further investigated the effects of PA, demonstrating that PA significantly decreased the number of neurons, reduced varicosities and restored the expression of SP and ChAT to normal levels. These findings suggested that PA might restore the local imbalance of neurotransmitters in the colonic myenteric plexus.

Our rat model of IBS-D was optimized through wrap-restraint stress as a single inducing factor. After 2 weeks, the IBS-D rats were characterized by an increased defecation area and augmented AWR responses to rectal distention, mimicking some clinical traits of IBS-D. This model can be reproducible and could therefore be utilized to investigate IBS-D and the mechanism of action of potential drugs. Interestingly, our study indicated that PA was beneficial in IBS-D rats by ameliorating intestinal transit and visceral hypersensitivity.

PA is an active ingredient of Pogostemonis Herba, an herb used for vomiting, diarrhea and other gastrointestinal diseases. Studies have shown that patchouli alcohol extracted from patchouli can produce antifungal and anti-inflammatory pharmacological effects, enhance the body’s resistance; PA also has a bidirectional effect on the stomach and intestines, promote gastric acid secretion, enhance gastrointestinal activity, regulate digestive function, while calming the smooth muscle of the gastrointestinal tract, and alleviate the spasmodic contraction of the gastrointestinal tract caused by irritating substances; Reduce the activity of enzymes that reflect gastrointestinal inflammation, prevent ulcerative gastroenteritis, protect the gastrointestinal mucosal barrier. (Chen et al., 1998; Hu et al., 2017; Liu et al., 2017). Previously, we showed that PA exerted an inhibitory effect on the spontaneous contraction of the colonic longitudinal smooth muscle in IBS-D rats (Sah et al., 2011). In this study, we showed that PA can downregulate the expression of ChAT by influencing proximal and distal colonic myenteric neurons.

At present, many animal models of IBS-D have been studied. Different animal models may reflect several pathological aspects of the disease, resulting in heterogeneous and inconsistent results. For example, the proportion of ChAT-immunoreactive neurons was significantly increased in the model of water avoidance stress (MAS), compared with controls. At the same time, no difference was found regarding the total number of neurons (Aubert et al., 2019).

The IBS-D disorder is closely related to an abnormal activation and proliferation of gastrointestinal neurons. Recent studies have demonstrated that the proliferation of gastrointestinal neurons is finely balanced. On the one hand, apoptotic neurons are efficiently removed by macrophages. On the other hand, the homeostasis of gastrointestinal neurons mainly depends on enteric glial cells with neuronal stem/progenitor properties (ENPCs). The enteric glial cells interact with neurons and participate in the regulation of gastrointestinal motility (Rao et al., 2017). In the gastrointestinal tract of mice, ENPCs can replace about 90% of neurons in around 2 weeks. Selective knockdown of PTEN gene in glial cells contributed to the regeneration of neurons (Kulkarni et al., 2017). If nestin and glial cells were selectively knocked out, the neurons proliferated (Kulkarni et al., 2017). In a rat model of constipation irritable bowel syndrome (IBS-C), the total number of neurons and the subtypes of excitatory and inhibitory neurons changed significantly. The total number of neurons per high-power field increased, while the proportion of cholinergic acetyltransferase immunoreactive neurons and activated vasoactive intestinal peptide positive neurons decreased. The proportion of NOS positive neurons increased, suggesting that these changes might be relevant in the pathogenesis of IBS-C (Fei et al., 2018).

The intestinal neurons, along with the microbiota, immune and glial cells have a key role in IBS-D (Collins et al., 2014; Neunlist et al., 2014; Sharkey, 2015; Veiga-Fernandes & Pachnis, 2017; Yoo & Mazmanian, 2017; Chandrasekharan et al., 2019). In a series of recent studies, the mouse and human gut advanced our understanding of the ENS (Amit et al., 2018; Drokhlyansky et al., 2020; Elmentaite et al., 2021; May-Zhang et al., 2021). A single-cell transcriptome analysis revealed different myenteric neuron classes in the small intestine of the mouse (Morarach et al., 2021). A comprehensive atlas of the cellular landscape across the human intestine was suggested by different studies (Elmentaite et al., 2021; Holloway et al., 2021). Another research provided new compelling evidence revealing the role of the ENS on immune cells. IL-6 secreted by enteric neurons was able to influence the number and phenotypes of regulatory T cells, which in turn modulated the structure and activity of ENS (Yan et al., 2021). In the future, the role of ENS in IBS-D will be further clarified. The use of more advanced technology such as transcriptomics and neuroimmunology will shed more light on enteric neural circuits and their relevance in gut motility, helping us to explore novel strategies to prevent or treat gastrointestinal diseases.

## Data Availability

The original contributions presented in the study are included in the article/supplementary material, further inquiries can be directed to the corresponding author.
