# Corrigendum: Patchouli alcohol improved diarrhea-predominant irritable bowel syndrome by regulating excitatory neurotransmission in the myenteric plexus of rats

**DOI:** 10.3389/fphar.2024.1394753

**Published:** 2024-03-15

**Authors:** Wanyu Chen, Lu Liao, Zitong Huang, Yulin Lu, Yukang Lin, Ying Pei, Shulin Yi, Chen Huang, Hongying Cao, Bo Tan

**Affiliations:** ^1^ Research Centre of Basic Intergrative Medicine, School of Basic Medical Sciences, Guangzhou University of Chinese Medicine, Guangzhou, China; ^2^ Shenzhen Hospital of Shanghai University of Traditional Chinese Medicine, Guangzhou, China; ^3^ College of Integrated Chinese and Western Medicine, Hunan University of Chinese Medicine, Changsha, Hunan, China; ^4^ School of Chinese Materia Medica, Guangzhou University of Chinese Medicine, Guangzhou, China

**Keywords:** patchouli alcohol, irritable bowel syndrome with diarrhea, colonic longitudinal muscle myenteric plexus, excitatory neurons, intestinal motility

In the published article, there was an error in the **Funding** statement, which incorrectly stated: “This work was funded by National Natural Science Foundation of China (grant number 8197141628).” The correct **Funding** statement appears below.

