# Supplementary material for: Patchouli alcohol improved diarrhea-predominant irritable bowel syndrome by regulating excitatory neurotransmission in the myenteric plexus of rats
Source: Front Pharmacol. 2022 Nov 14;13:943119. doi: 10.3389/fphar.2022.943119 (PMC9703083; doi:10.3389/fphar.2022.943119)
Supplement: Supplementary file 1 [file DataSheet1.PDF]

1. Fecal areas（mm2）

Tukey's multiple comparisons test	Mean Diff.	95.00% CI of diff.	Below threshold?	Summary	Adjusted P Value	
Control vs. IBS-D	-228.3	-357.2 to -99.33	Yes	**	0.0046	A-B
Control vs. PA（5mg/kg）	34.35	-94.60 to 163.3	No	ns	0.8157	A-C
Control vs. PA（10mg/kg）	60.19	-68.76 to 189.1	No	ns	0.4306	A-D
Control vs. PA（20mg/kg）	79.33	-49.61 to 208.3	No	ns	0.2354	A-E
IBS-D vs. PA（5mg/kg）	262.6	133.7 to 391.6	Yes	**	0.0024	B-C
IBS-D vs. PA（10mg/kg）	288.5	159.5 to 417.4	Yes	**	0.0016	B-D
IBS-D vs. PA（20mg/kg）	307.6	178.7 to 436.6	Yes	**	0.0011	B-E
PA（5mg/kg） vs. PA（10mg/kg）	25.84	-103.1 to 154.8	No	ns	0.9188	C-D
PA（5mg/kg） vs. PA（20mg/kg）	44.99	-83.96 to 173.9	No	ns	0.6532	C-E
PA（10mg/kg） vs. PA（20mg/kg）	19.15	-109.8 to 148.1	No	ns	0.9698	D-E

2. Anti-ChAT/Anti-HuC/D (%)

Tukey's multiple comparisons test	Mean Diff.	95.00% CI of diff.	Below threshold?	Summary	Adjusted P Value	
Control vs. IBS-D	-12.60	-19.56 to -5.638	Yes	**	0.0041	A-B
Control vs. PA（5mg/kg）	-6.328	-13.29 to 0.6306	No	ns	0.0707	A-C
Control vs. PA（10mg/kg）	0.1675	-6.791 to 7.126	No	ns	>0.9999	A-D
Control vs. PA（20mg/kg）	3.009	-3.950 to 9.968	No	ns	0.4904	A-E
IBS-D vs. PA（5mg/kg）	6.268	-0.6906 to 13.23	No	ns	0.0731	B-C
IBS-D vs. PA（10mg/kg）	12.76	5.805 to 19.72	Yes	**	0.0039	B-D
IBS-D vs. PA（20mg/kg）	15.61	8.647 to 22.56	Yes	**	0.0015	B-E
PA（5mg/kg） vs. PA（10mg/kg）	6.496	-0.4631 to 13.45	No	ns	0.0644	C-D
PA（5mg/kg） vs. PA（20mg/kg）	9.338	2.379 to 16.30	Yes	*	0.0154	C-E
PA（10mg/kg） vs. PA（20mg/kg）	2.842	-4.117 to 9.801	No	ns	0.5352	D-E

3. ChAT mRNA (AU)

Tukey's multiple comparisons test	Mean Diff.	95.00% CI of diff.	Below threshold?	Summary	Adjusted P Value	
Control vs. IBS-D	-0.6027	-1.127 to -0.07838	Yes	*	0.0292	A-B
Control vs. PA（5mg/kg）	-0.08279	-0.6071 to 0.4415	No	ns	0.9628	A-C
Control vs. PA（10mg/kg）	0.1081	-0.4162 to 0.6324	No	ns	0.9114	A-D
Control vs. PA（20mg/kg）	0.03991	-0.4844 to 0.5642	No	ns	0.9974	A-E
IBS-D vs. PA（5mg/kg）	0.5199	-0.004407 to 1.044	No	ns	0.0516	B-C
IBS-D vs. PA（10mg/kg）	0.7108	0.1865 to 1.235	Yes	*	0.0148	B-D
IBS-D vs. PA（20mg/kg）	0.6426	0.1183 to 1.167	Yes	*	0.0225	B-E
PA（5mg/kg） vs. PA（10mg/kg）	0.1909	-0.3335 to 0.7152	No	ns	0.6225	C-D
PA（5mg/kg） vs. PA（20mg/kg）	0.1227	-0.4016 to 0.6470	No	ns	0.8709	C-E
PA（10mg/kg） vs. PA（20mg/kg）	-0.06817	-0.5925 to 0.4562	No	ns	0.9811	D-E

4. ChAT/β-actin

Tukey's multiple comparisons test	Mean Diff.	95.00% CI of diff.	Below threshold?	Summary	Adjusted P Value	
Control vs. IBS-D	-0.4840	-0.7699 to -0.1982	Yes	**	0.0056	A-B
Control vs. PA（5mg/kg）	-0.1366	-0.4225 to 0.1493	No	ns	0.4123	A-C
Control vs. PA（10mg/kg）	-0.03312	-0.3190 to 0.2528	No	ns	0.9876	A-D
Control vs. PA（20mg/kg）	-0.03029	-0.3162 to 0.2556	No	ns	0.9911	A-E
IBS-D vs. PA（5mg/kg）	0.3474	0.06152 to 0.6333	Yes	*	0.0233	B-C
IBS-D vs. PA（10mg/kg）	0.4509	0.1650 to 0.7368	Yes	**	0.0077	B-D
IBS-D vs. PA（20mg/kg）	0.4538	0.1679 to 0.7397	Yes	**	0.0075	B-E
PA（5mg/kg） vs. PA（10mg/kg）	0.1035	-0.1824 to 0.3894	No	ns	0.6264	C-D
PA（5mg/kg） vs. PA（20mg/kg）	0.1063	-0.1796 to 0.3922	No	ns	0.6065	C-E
PA（10mg/kg） vs. PA（20mg/kg）	0.002830	-0.2831 to 0.2887	No	ns	>0.9999	D-E

5. SP-IR varicosity area (μm2)

Tukey's multiple comparisons test	Mean Diff.	95.00% CI of diff.	Below threshold?	Summary	Adjusted P Value	
Control vs. IBS-D	-0.3912	-0.7203 to -0.06202	Yes	*	0.0255	A-B
Control vs. PA（5mg/kg）	0.3266	-0.002574 to 0.6557	No	ns	0.0515	A-C
Control vs. PA（10mg/kg）	0.8050	0.4759 to 1.134	Yes	**	0.0010	A-D
Control vs. PA（20mg/kg）	0.6840	0.3549 to 1.013	Yes	**	0.0022	A-E
IBS-D vs. PA（5mg/kg）	0.7177	0.3886 to 1.047	Yes	**	0.0017	B-C
IBS-D vs. PA（10mg/kg）	1.196	0.8671 to 1.525	Yes	***	0.0001	B-D
IBS-D vs. PA（20mg/kg）	1.075	0.7460 to 1.404	Yes	***	0.0003	B-E
PA（5mg/kg） vs. PA（10mg/kg）	0.4785	0.1493 to 0.8076	Yes	*	0.0110	C-D
PA（5mg/kg） vs. PA（20mg/kg）	0.3575	0.02832 to 0.6866	Yes	*	0.0365	C-E
PA（10mg/kg） vs. PA（20mg/kg）	-0.1210	-0.4501 to 0.2081	No	ns	0.6151	D-E

6. SP mRNA (AU)

Tukey's multiple comparisons test	Mean Diff.	95.00% CI of diff.	Below threshold?	Summary	Adjusted P Value	
Control vs. IBS-D	-1.090	-1.818 to -0.3610	Yes	**	0.0097	A-B
Control vs. PA（5mg/kg）	-0.1650	-0.8936 to 0.5636	No	ns	0.8826	A-C
Control vs. PA（10mg/kg）	-0.2089	-0.9375 to 0.5198	No	ns	0.7777	A-D
Control vs. PA（20mg/kg）	-0.1042	-0.8328 to 0.6244	No	ns	0.9736	A-E
IBS-D vs. PA（5mg/kg）	0.9246	0.1960 to 1.653	Yes	*	0.0195	B-C
IBS-D vs. PA（10mg/kg）	0.8807	0.1521 to 1.609	Yes	*	0.0238	B-D
IBS-D vs. PA（20mg/kg）	0.9854	0.2568 to 1.714	Yes	*	0.0149	B-E
PA（5mg/kg） vs. PA（10mg/kg）	-0.04388	-0.7725 to 0.6847	No	ns	0.9990	C-D
PA（5mg/kg） vs. PA（20mg/kg）	0.06078	-0.6678 to 0.7894	No	ns	0.9964	C-E
PA（10mg/kg） vs. PA（20mg/kg）	0.1047	-0.6240 to 0.8333	No	ns	0.9731	D-E
